# Stoichiometry modulates the optoelectronic functionality of zinc phosphide (Zn_3−*x*_P_2+*x*_)†

**DOI:** 10.1039/d2fd00055e

**Published:** 2022-04-11

**Authors:** Elias Z. Stutz, Santhanu P. Ramanandan, Mischa Flór, Rajrupa Paul, Mahdi Zamani, Simon Escobar Steinvall, Diego Armando Sandoval Salaiza, Clàudia Xifra Montesinos, Maria Chiara Spadaro, Jean-Baptiste Leran, Alexander P. Litvinchuk, Jordi Arbiol, Anna Fontcuberta i Morral, Mirjana Dimitrievska

**Affiliations:** Laboratory of Semiconductor Materials, Institute of Materials, Faculty of Engineering, Ecole Polytechnique Fédérale de Lausanne 1015 Lausanne Switzerland mirjana.dimitrievska@epfl.ch; Catalan Institute of Nanoscience and Nanotechnology (ICN2), CSIC, BIST, Campus UAB Bellaterra Barcelona Catalonia Spain; Texas Center for Superconductivity and Department of Physics, University of Houston Houston Texas 77204-5002 USA; ICREA Pg. Lluís Companys 23 Barcelona Catalonia Spain; Institute of Physics, Faculty of Basic Sciences, Ecole Polytechnique Fédérale de Lausanne 1015 Lausanne Switzerland

## Abstract

Predictive synthesis–structure–property relationships are at the core of materials design for novel applications. In this regard, correlations between the compositional stoichiometry variations and functional properties are essential for enhancing the performance of devices based on these materials. In this work, we investigate the effect of stoichiometry variations and defects on the structural and optoelectronic properties of monocrystalline zinc phosphide (Zn_3_P_2_), a promising compound for photovoltaic applications. We use experimental methods, such as electron and X-ray diffraction and Raman spectroscopy, along with density functional theory calculations, to showcase the favorable creation of P interstitial defects over Zn vacancies in P-rich and Zn-poor compositional regions. Photoluminescence and absorption measurements show that these defects create additional energy levels at about 180 meV above the valence band. Furthermore, they lead to the narrowing of the bandgap, due to the creation of band tails in the region of around 10–20 meV above the valence and below the conduction band. The ability of zinc phosphide to form off-stoichiometric compounds provides a new promising opportunity for tunable functionality that benefits applications. In that regard, this study is crucial for the further development of zinc phosphide and its application in optoelectronic and photovoltaic devices, and should pave the way for defect engineering in this kind of material.

## Abstract 2

Zinc phosphide (Zn_3_P_2_) is a promising material for photovoltaic applications. Here, we investigate the effect of stoichiometry variations and defects on the structural and optoelectronic properties of monocrystalline Zn_3_P_2_.

## Graphical abstract

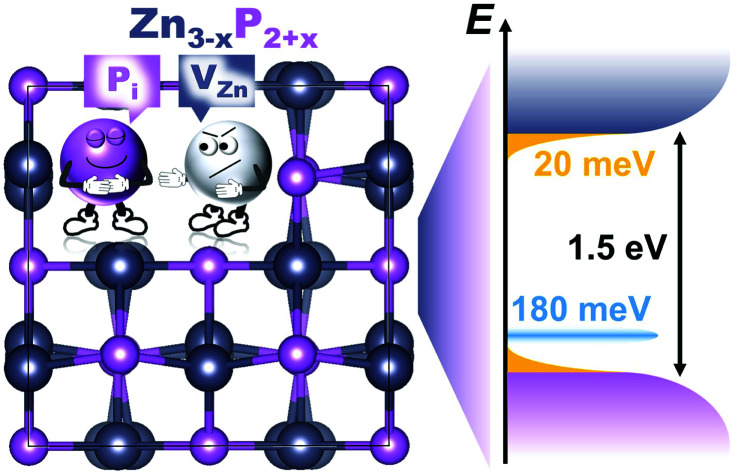

## Introduction

The effective design of materials with enhanced functional properties for novel applications requires the development of predictive synthesis–structure–property relationships. This can become very challenging in practice as many promising materials deviate from the ideal ordered stoichiometric compounds, exhibiting complexities such as off-stoichiometry, disorder, and inhomogeneities. Such non-ideal complications can have a profound effect on materials’ properties and applications, due to the additional degrees-of-freedom which can lead to variations in the charge and bond strength in between the atoms, as well as the creation of compositional defects and even secondary phases.

Semiconductor materials grown under nonequilibrium conditions form either stoichiometric compounds with the additional formation of secondary phases, or off-stoichiometric compounds with an increased concentration of defects. In many cases, these additions prove to be detrimental and preclude semiconductor applications. However, for some materials, off-stoichiometric growth provides a new dimension, leading to opportunities for tunable functionality that benefit applications.

Zinc phosphide (Zn_3_P_2_) is one such example. Zn_3_P_2_ has a relatively large and complex tetragonally distorted fluorite structure characterized by alternating layers of cations (Zn) and anions (P) along the [001] direction (*c*-axis).^1^ Interestingly for this structure, one quarter of the sites in the Zn-substructure are vacant. This ensures the phase stability of a substantial range of compositions and provides ample opportunities for material design by variations in stoichiometry.

Intrinsically, Zn_3_P_2_ is a semiconductor with a direct band gap of 1.5 eV, a high absorption coefficient of 10^4^ to 10^5^ cm^−1^, and long carrier diffusion lengths of ∼10 μm, which makes it a very promising material for photovoltaic (PV) applications.^2–15^ Additionally, it is constituted from sustainable earth-abundant elements. While Zn_3_P_2_ has many of the bulk properties required to be a high efficiency solar cell absorber, the current record efficiency of 6% is still well below the theoretical limit (>30%).^14^

Several main challenges have been identified as major roadblocks for achieving high efficiency devices. One is related to the synthesis of highly crystalline zinc phosphide on commercially available substrates. This has been solved recently by employing innovative synthesis methods, such as selective area epitaxy and van der Waals epitaxy on graphene.^16–19^ However, fine-tuning of the material’s functional properties, as well as optimizing the device structure, still remains a challenge.

Utilizing the vacant sites in the Zn_3_P_2_ structure for both intrinsic and extrinsic defect engineering is an effective way of obtaining desirable optoelectronic properties which will lead to high efficiency devices. This is why combinatorial studies, in which the effect of stoichiometric variations on the fundamental properties of Zn_3_P_2_ are explored, are crucial for further advancement of the PV technology based on this material.

In the 1980s, two important studies correlating the effect of stoichiometry on the carrier concentration of Zn_3_P_2_ were published.^20,21^ In the first one, Catalano *et al.*^20^ explored polycrystalline Zn_3_P_2_ thin films which were annealed over a range of equilibrium vapor compositions and temperatures. They observed an increase in the hole carrier concentration from 10^13^ to 10^16^ cm^−3^ with the increase in P pressure. Several years later, Fuke *et al.*^21^ investigated close to stoichiometric and slightly Zn-rich Zn_3_P_2_ ingot crystals and obtained similar trends, where the hole carrier concentration increased from 10^15^ to 10^16^ upon the increase in P concentration. Both of these works postulated that an increase in the concentration of P interstitial (P_i_) defects acting as acceptors is responsible for the increase in the carrier concentration. This was later on confirmed by density functional theory (DFT) calculations on defect dynamics in Zn_3_P_2_, which showed that P interstitials (P_i_) and Zn vacancies (V_Zn_) have the lowest formation energies in Zn_3_P_2_ grown under Zn-poor and P-rich conditions.^22,23^

Additionally, a few other works have investigated the effect of the incident elemental flux ratios during growth, the P/Zn flux ratio, on the formation of zinc phosphide thin films and nanowires.^17,18,24^ However, systematic studies on correlating the stoichiometry variations in Zn_3_P_2_ and defects with the functional properties are still scarce, and therefore very necessary.

In this work, we provide new insights on the influence of stoichiometry variations on the properties of monocrystalline Zn_3_P_2_ thin films. We demonstrate how the structural and functional properties respond to deviations from stoichiometry, whereby we give a tangible outlook for the defect engineering of such materials.

## Experimental methods

### Sample preparation

Monocrystalline Zn_3_P_2_ thin films were grown by Veeco GENxplor molecular-beam epitaxy, operating with separate Zn and P_2_ sources, on InP (100) substrates. Substrate preparation included two steps of degassing for 2 h at 150 °C and 300 °C and a third stage at 580 °C under P_2_ equivalent beam pressures > 1 × 10^−6^ Torr, with a duration of 10 min. The growth methods are explained in detail in ref. 18.

### Compositional characterization

Rutherford backscattering spectrometry (RBS) measurements, carried out by EAG Laboratories, were taken with a nearly-normally-incident beam of 2.275 MeV alpha particles. The normal detector angle collected particles scattered by 160° and the grazing detector was set at 104°. Assumptions of 6.61 × 10^22^ atoms per cm^3^ in the zinc phosphide layer and 5.26 × 10^22^ atoms per cm^3^ in the indium phosphide were used, and the atomic concentration uncertainty is ±1%. High angle annular dark field scanning transmission electron microscopy (HAADF STEM) images and energy dispersive X-ray spectroscopy (EDX) elemental maps were collected using a FEI Talos transmission electron microscope (TEM) operated at 200 kV.

### Structural characterization

X-ray diffraction measurements were performed using a Panalytical Empyrean diffractometer operating in Gonio scan configuration with a copper (Kα) X-ray source with a 1.54 Å wavelength, operating at 35 keV and 40 mA. Selected area diffraction patterns (SAED) were collected using a FEI Talos TEM operated at 200 kV.

### Angle resolved polarized Raman spectroscopy

Polarized Raman spectroscopy was realized in the backscattering configuration at 12 K. The 488 nm line of a Coherent sapphire optically pumped semiconductor laser was used for excitation. The beam was focused on the sample with a microscope objective with a numerical aperture of 0.75, resulting in a 1 μm diameter spot, reaching a radiant power of the order of 700 μW. The incident flux was controlled by combining a half-waveplate and a polarization beam-splitter. The incident polarization was controlled with a subsequent half-waveplate. The backscattered light went through a linear polarizer, and a last half-waveplate directed the polarization parallel to the entrance slit of a TriVista triple spectrometer with 900, 900 and 1800 cm^−1^ gratings in the subtractive mode and a Princeton Instrument liquid nitrogen cooled multichannel CCD PyLoN camera. The polarization direction was described with respect to a reference direction on the setup and moved in the *xy* crystallographic plane of the sample. All the spectra were calibrated based on the reference sulfur Raman spectrum.

### Photoluminescence spectroscopy

The micro-photoluminescence measurements were acquired in back-scattering geometry at 30 K using the 488 nm line of a Coherent sapphire laser and an Andor iDus DV420A-OE detector. A microscope objective with a numerical aperture of 0.75 was used to focus the light onto the sample to a spot with a diameter of about 1 μm. Radiant flux of approximately 1 mW with one hundred accumulations of 1 s were used. The photoluminescence spectra were corrected for the spectral sensitivity of the detection system and transformed to the energy scale with the Jacobian transformation. Parasitic features of the spectra, such as cosmic rays and a diffracted peak of the laser at 1.27 eV, were replaced with artificial data points for clarity.

### Ellipsometry measurements

Ellipsometry measurements were done on a Semilab SE-2000 spectroscopic ellipsometer in the energy range from 1.25 to 4 eV. The ellipsometry model was created and fitted using Semilab’s Spectroscopic Ellipsometry Analyzer software.

### Density functional theory calculations

The first-principles calculations of the electronic ground state of the tetragonally structured Zn_3_P_2_ were performed within the local density approximation using the Ceperly–Adler functional,^25,26^ as implemented in the CASTEP code.^27^ Norm-conserving pseudopotentials were used. The cutoff energy for the plane wave basis was set to 600 eV. A self-consistent field (SCF) tolerance better than 10^−7^ eV per atom and a phonon SCF threshold of 10^−12^ eV per atom were imposed. Prior to performing calculations, the structure was relaxed so that forces on atoms in the equilibrium position did not exceed 2 meV Å^−1^ and the residual stress was below 5 MPa. Experimentally determined lattice parameters from ref. 1 were used as a starting point. An integration over the Brillouin zone was performed over a 3 × 3 × 2 Monkhorst–Pack grid in reciprocal space.

## Results

### Compositional and morphological characterization of zinc phosphide thin films

**Fig. 1:**
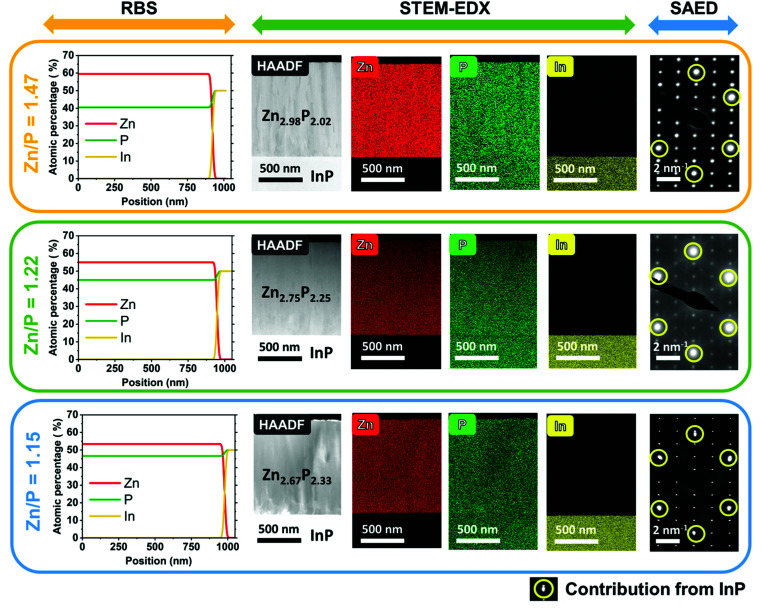
Compositional and morphological characterization of monocrystalline zinc phosphide thin films preformed using RBS, STEM-EDX and SAED measurements. RBS measurements were used to calculate the integral composition of each sample. The top panel corresponds to the close to stoichiometric thin film with a composition of Zn_2.98_P_2.02_ (Zn/P = 1.47), the middle panel corresponds to the slightly P-rich thin film with a composition of Zn_2.75_P_2.25_ (Zn/P = 1.22), and the bottom panel displays the most P-rich film with a composition of Zn_2.67_P_2.33_ (Zn/P = 1.15). Note that the circled diffraction spots in the SAED patterns correspond to the contribution of the InP substrate, while all the other diffraction spots are correlated with the tetragonal structure (*P*4_2_/*nmc*) of zinc phosphide.

We start by providing evidence on the composition and crystallinity of the samples investigated in this study. Fig. 1 gives an overview of the compositional and morphological characterization of the three zinc phosphide thin films obtained by RBS, STEM-EDX, and SAED measurements.

The RBS analysis shows a uniform distribution of Zn and P along the depth profile of all three films. These measurements have been used to calculate the integral compositional ratios of Zn and P for each sample. The thin film shown in the top panel in Fig. 1 has a close to stoichiometric composition of Zn_2.98_P_2.02_ (Zn/P = 1.47), while the thin film in the middle panel in Fig. 1 has a higher concentration of P, resulting in an off-stoichiometry composition of Zn_2.75_P_2.25_ (Zn/P = 1.22). Finally, the thin film in the bottom panel in Fig. 1 is the most P-rich, with a calculated composition of Zn_2.67_P_2.33_ (Zn/P = 1.15).

The cross-sectional HAADF STEM images of the zinc phosphide thin films grown on InP (100) substrates are shown in the second column of Fig. 1. This is followed by the compositional maps of Zn, P and In elements obtained by STEM-EDX. A homogenous distribution of Zn and P with no detectable phase segregation or intermixing with indium (In), within the technique’s resolution, is observed across all thin films.

SAED patterns measured on the cross-sections of thin films show overlapped patterns of zinc phosphide and InP (the most-right column in Fig. 1) and confirm the monocrystalline nature of all the samples. More intense diffraction spots (circled in yellow color in Fig. 1) correspond to the InP substrate, while the less intense diffraction spots belong to the zinc phosphide phase. The InP spots overlap with some Zn_3_P_2_ spots for the samples with Zn/P = 1.47 and Zn/P = 1.22, while for the sample with Zn/P = 1.15, we can distinguish a slight splitting of the InP and the Zn_3_P_2_ spots even with the naked eye. The latter indicates lattice distortions in the Zn_3_P_2_ structure induced by the different stoichiometry. Calculations of atomic distances based on the SAED patterns corroborate the formation of the tetragonal (*P*4_2_/*nmc*) unit cell of zinc phosphide and the growth direction along the *c* crystal axis ([001]), which is perpendicular to the substrate.

### Structural characterization of zinc phosphide thin films

**Fig. 2:**
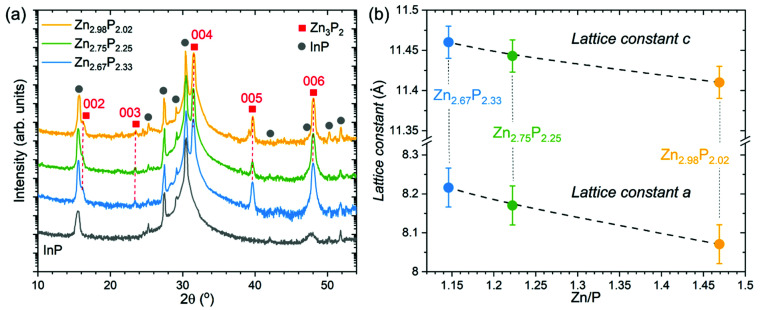
Structural analysis of the monocrystalline zinc phosphide thin films. (a) XRD patterns of three monocrystalline zinc phosphide thin films with various Zn/P compositions, along with the XRD pattern of the bare InP substrate measured under the same conditions. Gray circles denote reflections corresponding to the InP substrate, while red squares denote reflections belonging to the tetragonal structure (*P*4_2_/*nmc*) of zinc phosphide. (b) Compositional dependence of the lattice constants *a* and *c* calculated from the XRD and SAED measurements.

Structural characterization of the three zinc phosphide thin films was performed using XRD. Fig. 2a presents the measured XRD patterns of each sample, along with the XRD pattern of the bare InP substrate, which was used as a reference. Gray circles are employed to highlight the locations of the peaks stemming from the InP substrate. The other peaks in the measured patterns, labeled with red squares in Fig. 2a, are identified as reflections from {00*l*} (*l* = 2, 3, 4, 5 and 6) planes and belong to the tetragonal structure (*P*4_2_/*nmc*) of zinc phosphide. These results confirm the monocrystalline nature of all thin films, and the growth direction along the [001] axis, consistent with the TEM analysis.

The 2*θ* positions of the observed zinc phosphide reflections in the XRD patterns have been used to calculate the *c* lattice constant of the tetragonal unit cell. It should be noted that the orientation of the thin films does not allow direct determination of the *a* lattice constant from the XRD measurements. Therefore, we have used the SAED patterns to calculate the *c*/*a* ratio from the atomic distances. Utilizing the obtained *c* lattice constant from the XRD, and the *c*/*a* ratio from the SAED patterns, we were able to estimate the *a* lattice constant. An overview of the calculated structural parameters is given in Table 1, while the graphical dependence of the *a* and *c* lattice constants on the composition is presented in Fig. 2b. The lattice constants calculated for the stoichiometric composition are in good agreement with previously reported results.^1^

**Table 1:** Structural parameters of zinc phosphide thin films obtained from XRD and SAED measurements

Composition of zinc phosphide	Zn/P	Lattice constant *c* (Å) calculated from XRD	*c*/*a* calculated from SAED	Lattice constant *a* (Å) calculated from XRD and SAED
Zn_2.98_P_2.02_	1.47	11.41 ± 0.02	1.414 ± 0.015	8.07 ± 0.05
Zn_2.75_P_2.25_	1.22	11.44 ± 0.02	1.401 ± 0.015	8.17 ± 0.05
Zn_2.67_P_2.33_	1.15	11.46 ± 0.02	1.395 ± 0.015	8.22 ± 0.05

Elongation of the zinc phosphide tetragonal unit cell in both the *a* and *c* directions is observed upon the change from stoichiometric to P-rich compositions. It is also interesting to note that the extension of the unit cell along the *a* direction (0.15 Å or 1.9%) is higher than along the *c* direction (0.05 Å or 0.5%).

We will discuss in detail the origin of the lattice extension with changes in the composition of zinc phosphide in the Discussion section.

### Polarization Raman measurements on zinc phosphide thin films

Raman spectroscopy is a versatile tool for studying structure and composition in materials, as it provides information on phase,^28^ defects,^29–32^ inhomogeneities,^33^ and crystallinity.^34^ Compositionally induced defects, such as point defects or defect clusters in the form of vacancies, substitutionals and interstitials mostly preserve the overall crystal symmetry. This means that the Raman selection rules are preserved, and no changes in the frequency of the main Raman modes is expected. Defects could either lead to the breaking or creation of chemical bonds inside the lattice, which in turn change the values of the polarizability tensors elements related to the vibrational modes involving these bonds, and finally affect the intensity of the Raman peaks. Furthermore, some defects can lead to the creation of local vibrational modes, which result in the appearance of new peaks in the Raman spectrum.

Zinc phosphide has a very rich Raman spectrum with 39 modes (9 A_1g_, 10 B_1g_, 4 B_2g_ and 16 E_g_) in the frequency region lower than 400 cm^−1^.^35,36^ This makes the tracking of changes in the intensity of Raman modes caused by the presence of defects rather difficult, due to the strong overlap of the peaks. This challenge can be solved by utilizing angle resolved polarization Raman spectroscopy. Different polarization configurations allow the activation of only certain types of vibrational modes in the Raman spectrum. This means that the number of peaks in the Raman spectrum can be significantly reduced by performing measurements in the specifically selected polarization configuration.

Detailed calculations on the angular dependencies of the Raman mode intensities for tetragonal zinc phosphide, in parallel and perpendicular polarization measurement configurations on the (001) basal plane for various polarization angles *θ*, are given in ref. 35. Based on these, we have chosen the perpendicular polarization configuration with *θ* = π/4, which allows the activation of only B_1g_ modes in the Raman spectrum of zinc phosphide. Fig. S1† gives a schematic representation of the Raman polarization measurements performed in this case.

**Fig. 3:**
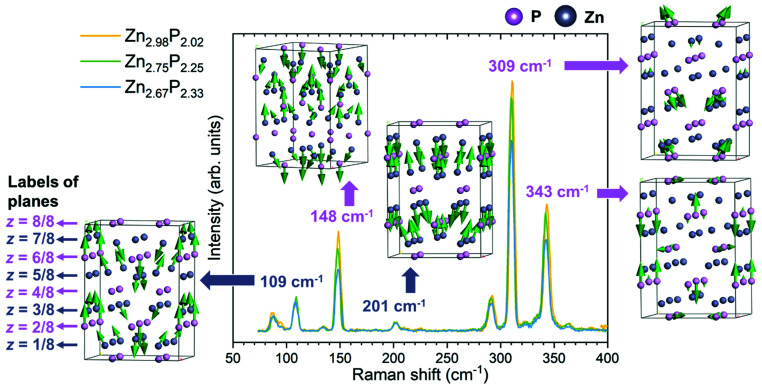
Polarization Raman spectroscopy. Polarization Raman spectra of the three monocrystalline zinc phosphide thin films with various compositions, along with five representative vibrational patterns of Raman modes at 109, 148, 201, 309 and 343 cm^−1^. Raman measurements were done in the perpendicular configuration with a polarization angle of *θ* = π/4 on a basal (001) plane at low temperature (12 K) and using 488 nm laser excitation. All thin films were oriented along the same [100] and [010] directions. These conditions allowed the activation of only the B_1g_ modes in the Raman spectra. Vibrational patterns were obtained from DFT calculations.

Fig. 3 presents the polarization Raman spectra obtained from three zinc phosphide thin films in the perpendicular configuration for a polarization angle of *θ* = π/4. All spectra were acquired at low temperature (12 K) and using 488 nm laser excitation. It should be noted that all three samples were aligned along the same [100] and [010] directions, which excludes the effect of the different grain orientation on the Raman spectra. This means that the changes observed in the Raman spectra are only caused by the variations in the composition of the zinc phosphide thin films, as all other measurement parameters have been kept the same.

Comparison of the three Raman spectra in Fig. 3 shows no significant changes in the positions or widths of the Raman peaks with the change in the composition of the thin films. However, changes in the intensity of the Raman peaks are observed. Some peaks (148, 290, 309 and 343 cm^−1^) decrease in intensity with the increase in P composition from stoichiometric to P-rich. Others (87, 109 and 201 cm^−1^) remain unchanged.

In order to better understand the change in the intensity of the peaks with the composition of zinc phosphide, representative vibrational patterns of Raman modes at 109, 148, 201, 309 and 343 cm^−1^ are illustrated in Fig. 3. These have been obtained from the DFT calculations. It can be noticed that all vibrational patterns of the B_1g_ modes are mostly characterized with the out of plane atomic vibrations, predominantly along the [001] direction.

The Raman mode at 201 cm^−1^ is characterized by the out of plane vibrations of all Zn atoms in the lattice. Similarly, the Raman modes at 109 and 148 cm^−1^ are mostly dominated by the Zn-atomic vibrations. However, they also involve vibrations of the P atoms located in the *z* = 3/8 and *z* = 6/8 planes for the 109 cm^−1^ mode, and the *z* = 4/8, *z* = 6/8 and *z* = 8/8 planes for the 148 cm^−1^ mode. On the other hand, the Raman modes at 309 and 343 cm^−1^ are characterized mostly by the vibrations of P atoms.

By comparing the different vibrational patterns, we deduce that the intensity changes in the Raman peaks with increasing the P composition should involve the vibrations of P atoms located in the *z* = 4/4 and *z* = 8/8 planes. The implications of this conclusion in terms of the location of the defects will be explained in the Discussion section.

### Optoelectronic characterization of zinc phosphide thin films

**Fig. 4:**
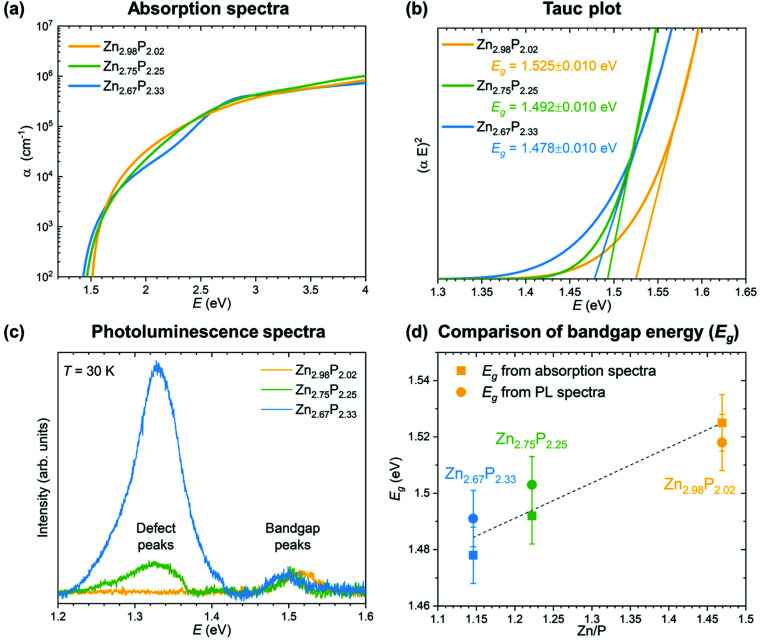
Optoelectronic characterization of the monocrystalline zinc phosphide thin films. (a) Comparison of the calculated absorption coefficients from the ellipsometry measurements for zinc phosphide thin films with various compositions. (b) Tauc plots used for the determination of the bandgap energy of zinc phosphide thin films. (c) Photoluminescence spectra of zinc phosphide thin films collected at 30 K with 488 nm laser excitation. (d) Compositional dependence of the bandgap energies calculated from absorption and PL measurements.

In order to evaluate the effect of variations in the composition on the optoelectronic properties of zinc phosphide, we have performed absorption and photoluminescence measurements. The results are shown in Fig. 4.

The absorption coefficients were calculated from the refractive indices and extinction coefficients obtained from ellipsometry measurements. The detailed procedure is explained in ref. 15. Fig. 4a shows a comparison of the absorption spectra from the three monocrystalline zinc phosphide thin films. All three absorption spectra follow a similar trend, with a steep increase in the absorption coefficient to 10^5^ cm^−1^ in the range from 1.5 to about 2 eV, after which the increment becomes more gradual until it reaches about 10^6^ cm^−1^ at around 4 eV. Some fluctuations in the shape of the absorption curve of the most P-rich thin film can be observed when compared to that of the stoichiometric zinc phosphide thin film. The spectral dependence of the absorption coefficients of the films is well described by the square-root dependence on energy *α* ∝ (*E* − *E*_g_)^0.5^, where *E*_g_ is the bandgap. This dependence arises from a parabolic approximation of the conduction and valence bands in the region close to *k* = 0 and agrees well with the absorption coefficient of most direct gap compound semiconductors.^37^

The bandgap values have been calculated from the Tauc plots for all three zinc phosphide films following the procedure explained in ref. 15. Fig. 4b presents the comparison of the Tauc plots, showing the dependence of (*αE*)^2^ on the *E* for each thin film. Linear extrapolation of the curves yields bandgap values in the range of 1.525 ± 0.010 eV for the near-stoichiometric sample to 1.478 ± 0.010 eV for the most P-rich sample. The bandgap values agree well with the previously reported results.^15,16,38,39^ The reduction in the bandgap energy with increasing P composition can be correlated with the increase in the Urbach energy (the width of the Urbach tail), which provides information on the electron–phonon interaction and structural disorder caused by the compositional deviation from the ideal stoichiometry.

The Urbach energy was calculated in the energy region just below the band edge, from the exponential dependence of the absorption coefficient on the photon energy (*α* = *a*_0_ exp[(*E* − *E*_0_)/*E*_U_], where *E*_0_ and *α*_0_ are the characteristic parameters of the material and *E*_U_ is the Urbach energy). Fig. S2(a–c) in the ESI† present the plots used for calculating the Urbach energy for all three zinc phosphide thin films. The results show an increase of the Urbach energy from 11 ± 3 meV for the near-stoichiometric sample to 43 ± 3 meV for the most P-rich sample. This dependence is shown in Fig. S2d in the ESI.† This points to the increase in structural disorder with the increase in P concentration, which leads to the long range electrostatic potential fluctuations, causing band tailing and effectively narrowing the bandgap, as observed by absorption measurements. It should be noted that the Urbach energy has not been previously reported for monocrystalline zinc phosphide, however a study by Zawawi *et al.*^40^ reports Urbach energies in the range of 200 to 330 meV for nanostructured zinc phosphide thin films. This is an order of magnitude higher than the Urbach energies from our samples, which is expected considering the significant improvement in the structural order of our thin films in comparison to that of the nanostructured thin films from ref. 40.

The results from the absorption measurements are also coherent with the photoluminescence (PL) measurements, which are shown in Fig. 4c. The PL spectra of the three zinc phosphide films were collected at 30 K using 488 nm laser excitation. No contribution from the InP substrate to the PL signal of the zinc phosphide thin films is observed upon comparison with the reference PL spectra of InP.

Two bands at around 1.32 and 1.50 eV dominate the PL response from the zinc phosphide samples. The lower intensity band at around 1.50 eV is observed in all three zinc phosphide thin films and, according to our recent study in ref. 39, is attributed to zone-center band-to-band electronic transitions. A progressive shift of this band’s position from 1.52 to 1.49 eV is seen with the increase in P composition. These positions agree well with the calculated bandgap energies from the absorption measurements, as shown in Fig. 4d, and further confirm this band’s attribution to band-to-band electronic transitions. This is consistent with the trend observed in cathodoluminescence measurements done on zinc phosphide nanowires.^24^

On the other hand, the band at around 1.32 eV progressively increases in intensity with the increase in P composition, with no significant shift in the position. According to the detailed temperature and power excitation dependence of this band in ref. 39, this transition is attributed to defect-related recombination. A higher concentration of compositionally induced defects is expected in more off-stoichiometric zinc phosphide samples, such as the ones with an excess of P composition. This creates more recombination centers, which essentially leads to the increase in the intensity of the defect band in the PL spectra with an increase in P composition, as observed in Fig. 4d.

More detailed discussion of the defect dynamics of zinc phosphide and their correlation with the optoelectronic properties will be provided in the next section.

## Discussion

**Fig. 5:**
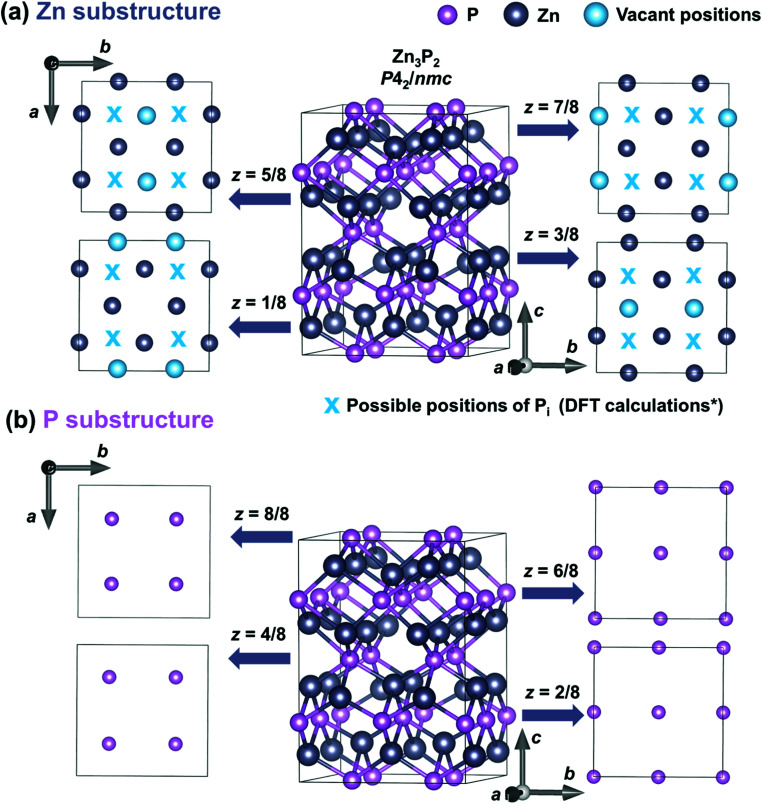
Crystal structure representation of a tetragonal Zn_3_P_2_ unit cell along different crystal planes. Representation of the Zn substructure (a) and P substructure (b) along the eight plane projections along the [001] direction (*z*-axis). Zn and P atoms are labeled with gray and pink circles, respectively. Blue circles denote the vacant positions in the Zn substructure, while blue “x” symbols show the positions of P_i_ defects obtained from the DFT calculations based on ref. 23 (marked with a * sign in the label).

Zinc phosphide has a tetragonal structure with alternating planes of Zn and P along the [001] direction. Fig. 5 shows the different plane projections of Zn and P substructures along the *c*-axis of the Zn_3_P_2_ structure. One quarter of the sites in the Zn-substructure (labeled with blue circles in Fig. 5a) are vacant, which provides plenty of opportunities for defect engineering in this material.

Two studies on defect dynamics in zinc phosphide have reported conflicting results in terms of the most probable defect type formation under Zn-poor and P-rich conditions.^22,23^ Both investigations have used DFT calculations with hybrid functional methods. Demers *et al.*^22^ have suggested that P interstitials (P_i_) are responsible for the p-type conductivity of zinc phosphide, due to their lowest formation energy. This is consistent with previous experimental observations of the tendency of conductivity for thin films obtained under a higher phosphorus partial pressure.^20,21^ On the other hand, according to calculations by Yin *et al.*,^23^ zinc vacancies (V_Zn_) have a lower formation energy than that of P_i_, suggesting that V_Zn_ are the main reason for the p-type conductivity.

Furthermore, it is postulated that P_i_ are single acceptors which occur in the voids in the Zn plane of atoms nearly equidistant from three Zn atoms and directly above a P atom displaced from the plane below.^23^ The approximate positions of these defects are labeled with a blue “x” in Fig. 5a. V_Zn_ are double acceptor defects which are formed on Zn sites.^22,23^

All zinc phosphide thin films used in this study exhibit an excess in the P composition. This is considered as the Zn-poor and P-rich compositional range, where the formations of P_i_ and V_Zn_ are most probable.

The elongation of the crystal lattice upon the addition of P, obtained from the XRD and SAED measurements, suggests that P_i_ interstitials are the dominating types of defects. This is based on the geometrical aspects of the insertion of additional atoms into the structure, which demands additional relaxation of the lattice. This is opposite to the formation of vacancies, which would more likely cause a reduction in the structural parameters.

The existence/incorporation of P_i_ is also supported by the Raman measurements. This is due to the reduction in the Raman peak intensity, which is only observed for modes involving the vibrations of P atoms located in the *z* = 4/8 and *z* = 8/8 planes. According to the DFT calculations,^23^ P_i_ defects, which are formed in the Zn plane, build strong covalent bonds with one of the host P atoms located in the *z* = 4/8 and *z* = 8/8 planes. This new covalent bonding modifies the vibrations involving these atoms, which finally results in the reduction of the intensity of the Raman peaks, as seen in Fig. 3. On the other hand, no changes in the Raman peak intensities of modes involving Zn atoms are observed. This further points to the concentration of V_Zn_ being significantly lower than that of P_i_.

Photoluminescence measurements point to the formation of a defect level at about 180 meV above the valence band. This is based on the assignment of the defect band at around 1.32 eV in the PL spectra. Additionally, absorption measurements point to the formation of band tails in the region of around 10–20 meV (*E*_U_/2) above the valence and below the conduction bands. The community is still divided on the attribution of these states to certain defect types. DFT study by Demers *et al.*,^22^ together with the transport measurements by Catalano *et al.*^20^ and Lombardi *et al.*,^41^ suggest the attribution of the level at 180 meV to V_Zn_, while the lower energy states in the band tails are attributed to P_i_. On the other hand, the DFT calculations by Yin *et al.*^23^ better correspond to the attribution of the level at 180 meV to P_i_, while the states in the band tails correlate well with the formation energies of V_Zn_. Considering these contradictions, further experimental and theoretical explorations of zinc phosphide defect states are planned in the future for better optimization of the material for optoelectronic applications.

Finally, assuming that all additional P atoms in the non-stoichiometric compositions lead to the formation of either only P_i_ or only V_Zn_, and using calculated lattice parameters from the XRD and SAED patterns, we can estimate the total concentration of structural defects present in our zinc phosphide films. Based on this, the defect concentration in all cases is of the order of 10^20^ to 10^21^ cm^−3^. This is several orders of magnitude higher than the measured carrier concentration in zinc phosphide, which is around 10^15^ to 10^16^ cm^−3^.^20,21^ Such a huge difference between the concentration of structural defects and carriers suggests that many of the structural defects are actually charge neutral and do not contribute to the conductivity process. This is supported by the DFT calculations from Demers *et al.*, which shows that the formation energies of the charge neutral P_i_ and V_Zn_ are among the lowest ones for the moderate to very p-type regimes of zinc phosphide.

## Conclusions

The effect of stoichiometry variations and compositionally induced defects on the structural and optoelectronic properties of monocrystalline zinc phosphide thin films was investigated in detail. Monocrystalline zinc phosphide thin films were grown on InP substrates using MBE. RBS measurements were used to determine the integral composition of three zinc phosphide samples, which were in the range from close to the stoichiometric composition of Zn_2.98_P_2.02_ (Zn/P = 1.47) to the P-rich composition of Zn_2.67_P_2.33_ (Zn/P = 1.15). Both RBS and EDX measurements have shown uniform distribution of elements, without any additional phase segregation or intermixing with In from the substrate.

XRD and SAED patterns confirmed the monocrystalline nature of all samples and allowed determination of the structural parameters. Elongation of the unit cell in all three crystallographic axis directions is observed for P-rich compositions, pointing to the formation of P_i_ as a dominating defect. This is also supported by the polarization Raman measurements and DFT calculations of vibrational patterns. Reduction in the Raman peak intensity is only observed for modes involving the vibrations of P atoms located in the *z* = 4/8 and *z* = 8/8 planes. On the other hand, no changes in the Raman peak intensities of modes involving Zn atoms are observed. This suggests that the concentration of V_Zn_ is significantly lower than that of P_i_.

Narrowing of the bandgap in the range from 1.525 ± 0.010 eV for the near-stoichiometric sample to 1.478 ± 0.010 eV for the most P-rich sample is observed from the absorption measurements. This is correlated with the increase of the Urbach energy from 11 ± 3 meV for the near-stoichiometric sample to 43 ± 3 meV for the most P-rich sample, showcasing the creation of band tails upon deviation from the stoichiometry.

Two bands at around 1.32 and 1.50 eV dominate the PL response from the zinc phosphide samples. These are attributed to the defect-related and band-to-band transitions, respectively. The position of the higher energy band progressively shifts from 1.52 to 1.49 eV with the increase in P composition, in agreement with the absorption measurements. The defect-related band increases in intensity upon the increase in P composition, with no obvious change in the position.

These results point to the creation of electronic levels in the bandgap at around 180 meV above the valence band. Additionally, band tails are observed in the region around 10–20 meV above the valence and below the conduction band. The presence of these levels can have a profound effect on the solar cell performance, as it can lead to the localization of photo-generated charge carriers or even charge trapping in the band tails. Further studies involving conductivity and time resolved photoluminescent measurements are needed in order to evaluate the effect of these states on the transport properties of zinc phosphide.

The present results offer significant insights into the structural and optoelectronic properties of zinc phosphide upon changes in the composition. The ability of zinc phosphide to form off-stoichiometric compounds provides a new opportunity for tuning the functional properties that benefit applications for optoelectronic and photovoltaic devices.

## Author contributions

M. D. and A. F. i. M. conceived the research and designed the experiments. M. Z. prepared the samples with the help of S. E. S., R. P. and J. B. L. M. D. performed the XRD measurements. E. Z. S. performed the photoluminescence and ellipsometry measurements. A. P. L. did the DFT calculations. S. P. R., C. X., M. C. S. and J. A. did the TEM analysis. M. F. and D. A. S. S. did the Raman measurements. M. D. analyzed the data, coordinated the simulations, and connected all results. M. D. wrote the paper with inputs from all the authors.

## Conflicts of interest

The authors declare that they have no competing financial interests.

## Supplementary Material

FD-239-D2FD00055E-s001
