# Supplementary material for: Stoichiometry modulates the optoelectronic functionality of zinc phosphide (Zn3−xP2+x)
Source: Faraday Discuss. 2022 Apr 11;239:202–18. doi: 10.1039/d2fd00055e (PMC9614774; doi:10.1039/d2fd00055e)
Supplement: FD-239-D2FD00055E-s001 [file FD-239-D2FD00055E-s001.pdf]

Electronic Supplementary Material (ESI) for Faraday Discussions.
This journal is © The Royal Society of Chemistry 2022

Supporting Information for:

Stoichiometry modulates the optoelectronic functionality of Zinc Phosphide ($Zn_{3-x}P_{2+x}$)

Elias Z. Stutz,[1] Santhanu P. Ramanandan,[1] Mischa Flor,[1] Rajrupa Paul,[1] Mahdi Zamani,[1] Simon Escobar Steinvall,[1] Diego Armando Sandoval Salaiza,[1] Claudia Xifra,[2] Maria Chiara Spadaro,[2] Jean-Baptiste Leran,[1] Alexander P. Litvinchuk,[3] Jordi Arbiol,[2,4] Anna Fontcuberta i Morral,[1,5] Mirjana Dimitrievska[1]*

1 - Laboratory of Semiconductor Materials, Institute of Materials, Faculty of Engineering, Ecole Polytechnique Fédérale de Lausanne, 1015 Lausanne, Switzerland.

2 – Catalan Institute of Nanoscience and Nanotechnology (ICN2), CSIC and BIST, Campus UAB, Bellaterra, Barcelona, Catalonia, Spain

3 - Texas Center for Superconductivity and Department of Physics, University of Houston, Houston, Texas 77204-5002, United States of America

4 - ICREA, Pg. Lluís Companys 23, Barcelona, Catalonia, Spain.

5 - Institute of Physics, Faculty of Basic Sciences, Ecole Polytechnique Fédérale de Lausanne, 1015 Lausanne, Switzerland.

* e-mail: mirjana.dimitrievska@epfl.ch

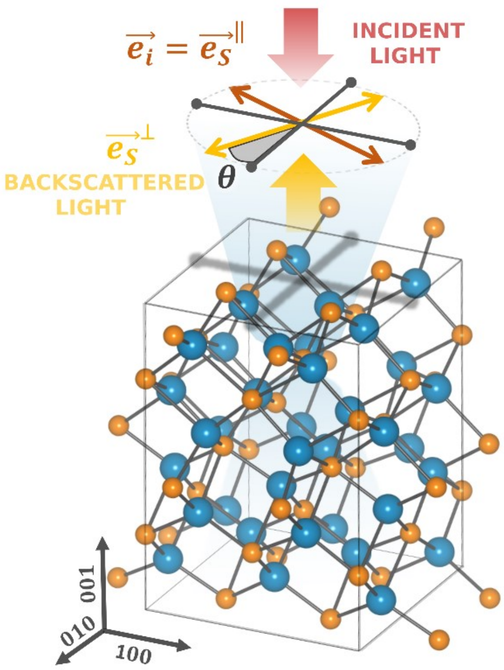

Figure S1. Schematic representation of the Raman scattering polarization measurements. Raman measurements are performed on the (001) basal plane with incident laser light (wide red arrow) projected along the -*z*-axis ([001] direction). The incident light polarization and the output polarization in perpendicular configuration are represented by the thin red and yellow arrows, respectively. The black arrows indicate the *x* and *y* axis of the crystal corresponding to [100] and [010] directions, respectively. The polarization angle $\theta = \pi/4$ was used for all three monocrystalline zinc phosphide thin films.

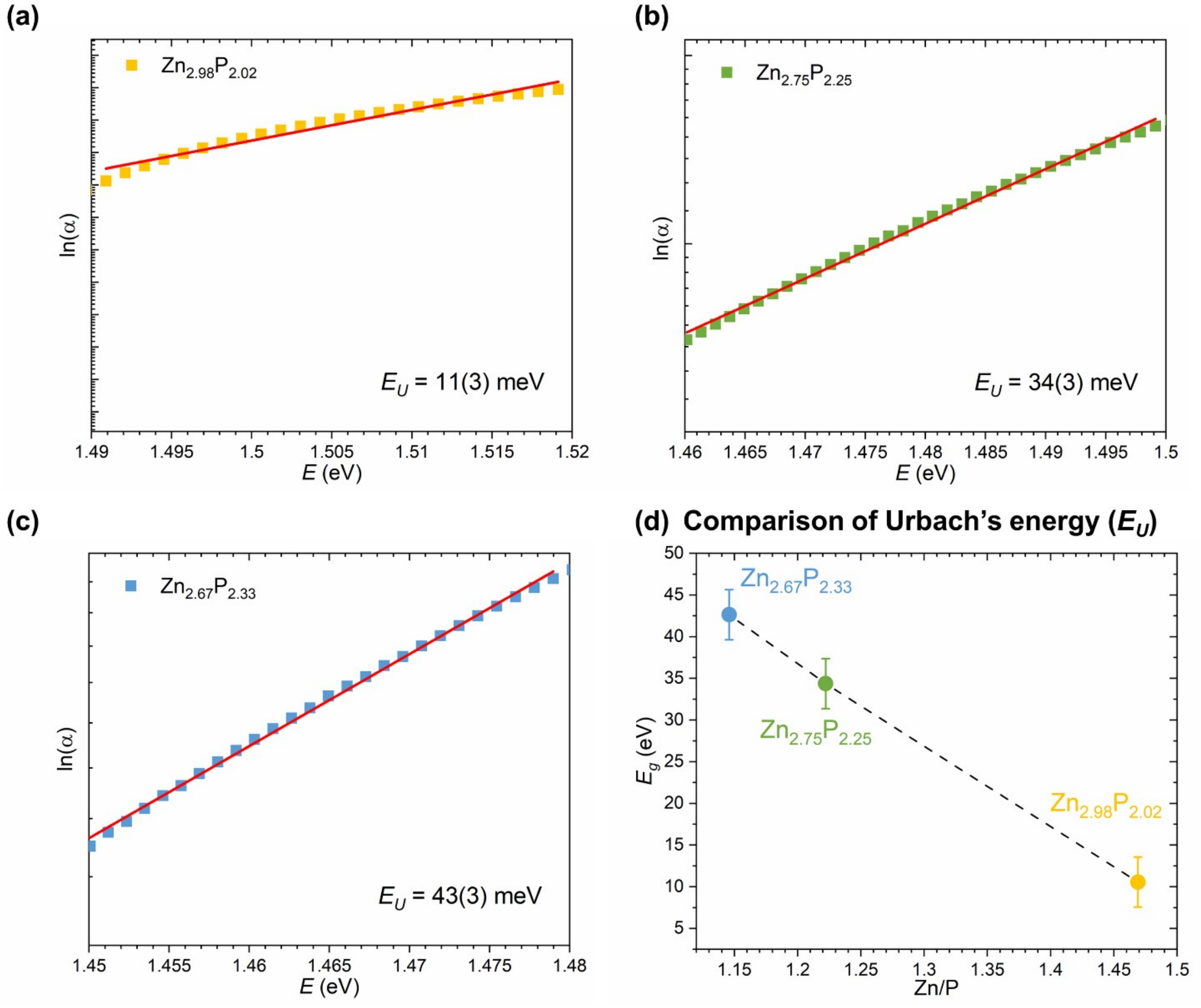

Figure S2. Urbach energy calculation from absorption measurements for samples with various compositions: (a) $Zn_{2.98}P_{2.02}$ (Zn/P = 1.47), (b) $Zn_{2.75}P_{2.25}$ (Zn/P = 1.22) and (c) $Zn_{2.67}P_{2.33}$ (Zn/P = 1.15). (d) Comparison of Urbach energy with composition.
